# Endoscopic Resection of Sinonasal Hemangiopericytoma following Preoperative Embolisation: A Case Report and Literature Review

**DOI:** 10.1155/2013/796713

**Published:** 2013-05-02

**Authors:** Georg J. Ledderose, Donata Gellrich, Markus Holtmannspötter, Andreas Leunig

**Affiliations:** ^1^Department of Otorhinolaryngology, Head and Neck Surgery, Ludwig Maximilian University Munich, Marchioninistraße 15, 81377 Munich, Germany; ^2^Institute for Neuroradiology, Ludwig Maximilian University Munich, Marchioninistraße 15, 81377 Munich, Germany; ^3^Center for Diagnostic Radiology, Department of Neuroradiology, Rigshospitalet, University of Copenhagen, Denmark; ^4^Center for Rhinology, Starnberg/Munich, Germany

## Abstract

*Objectives*. Hemangiopericytoma is a rare tumor entity deriving from pericytes. Less than 5% of hemangiopericytoma occur in the nasal cavity and are characterised by a rather benign nature with low tendency of metastasis. However, as the recurrence rate in the literature ranges from 9.5% to 50%—depending on the length of followup—a radical surgical resection is considered as the gold-standard treatment. Only a few years ago, a wide external approach, usually via lateral rhinotomy or Caldwell-Luc, was performed. Endoscopic techniques were regarded as appropriate for small low-vascularised tumors only. *Methods*. We present the case of a 64-year-old patient with an extended sinonasal hemangiopericytoma, who was successfully treated by an endoscopic controlled endonasal tumor resection after embolisation with Onyx. Further, to support the new treatment option, we review the literature concerning all features of sinonasal hemangiopericytomas and their therapeutical management. *Results/Conclusion*. Onyx, which has not been described in the context of hemangiopericytoma yet, is a very effective embolic agent for a preoperative embolisation of sinonasal hemangiopericytoma allowing a safe endoscopic surgery.

## 1. Introduction

Sinonasal hemangiopericytomas are a rare upper aerodigestive tract tumor deriving from perivascular modified smooth muscle cells. This vascular neoplasm firstly described in 1943 by Stout and Murray [1] may arise in any part of the body [2]; only 15%–30% are located in the head and neck region [3]. Of these, only 5% are found in the nasal cavity and paranasal sinus [4]. Hemangiopericytoma located in the nasal region is frequently characterized by a more benign nature with low tendency of metastasis [5]; however, sinonasal hemangiopericytoma exhibits a recurrence rate of approximately 25% [3].

In the last two decades, less than 250 cases have been published so that sinonasal hemangiopericytoma represents a rather rare tumor entity. A total of 57 patients out of the 250 analysed cases underwent an endoscopic tumor resection, of which 23 patients received a preoperative embolisation of the high vascularised hemangiopericytoma. 

Here, we review the relevant literature and report about a 64-year-old patient presenting with sinonasal hemangiopericytoma. We used a liquid embolic agent, consisting of an ethylene vinyl alcohol dissolved in the organic solvent dimethyl sulfoxide (DMSO) and containing tantalum powder for radiopacity (Onyx) and performed an endoscopically controlled resection. To our knowledge, this is the first report about preoperative embolisation of sinonasal hemangiopericytoma using this material.

## 2. Case Report

**Figure 1:**
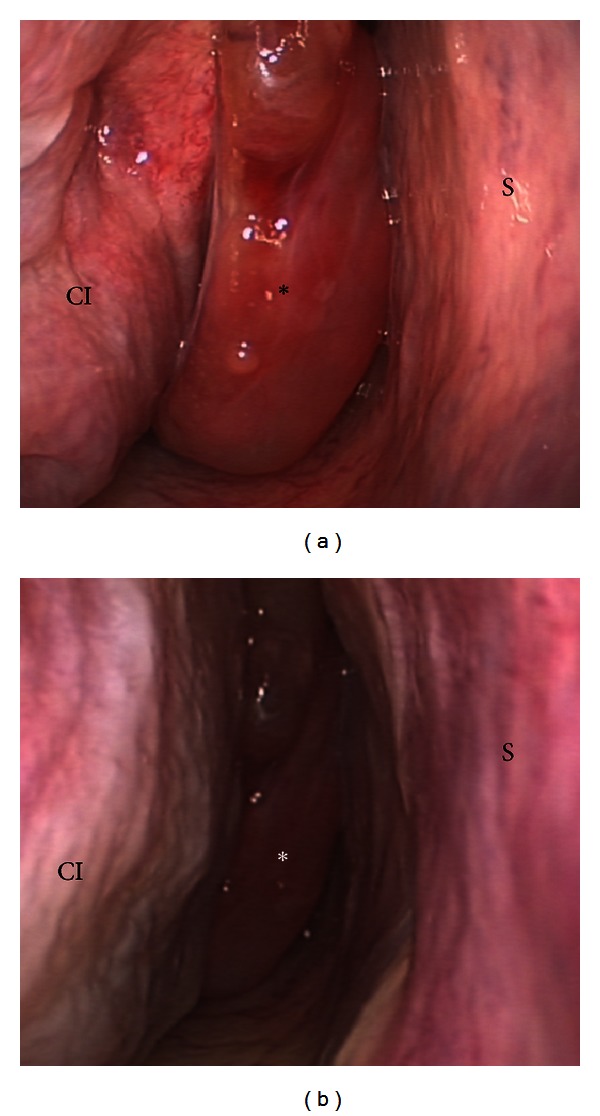
Endoscopic view of the operational field before surgery (a). After embolization, shrinking is clearly seen (b). Nasal septum (S), concha inferior (CI), concha media (CM), sphenoid sinus (SS), and tumor (*).

**Figure 2:**
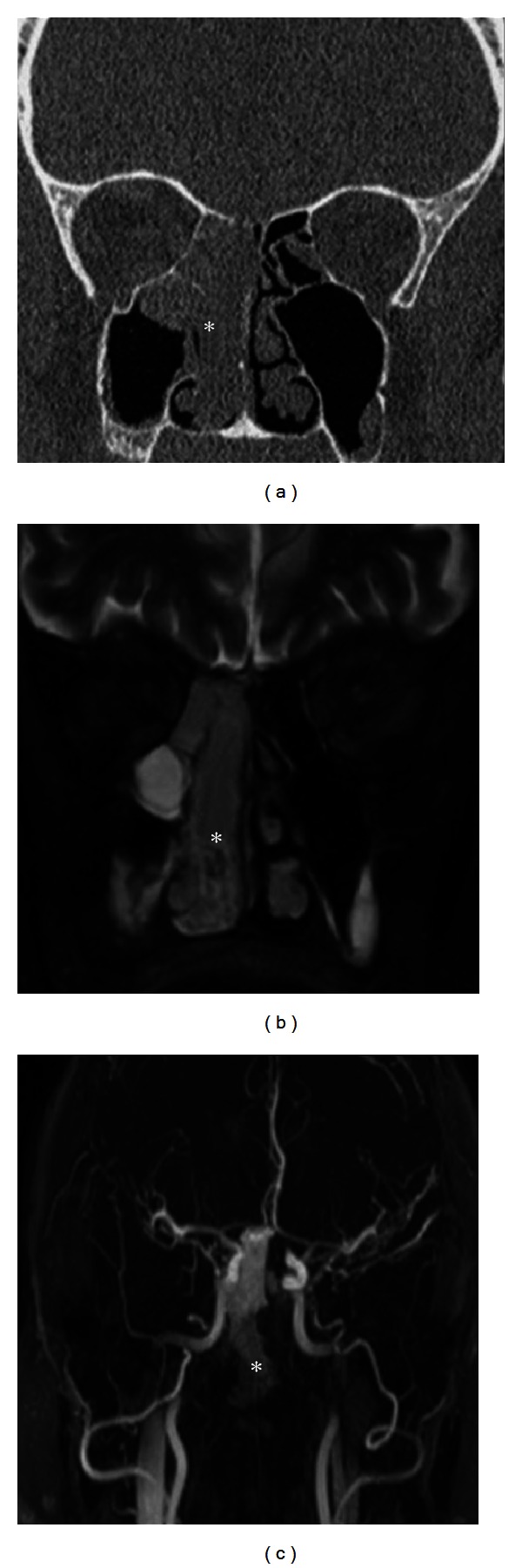
Radiologic findings of sinonasal hemangiopericytoma. CT scan (a), MRI scan (b), and MRI angiography (c). A circumscribed, submucosal, heterogeneous soft-tissue mass measuring 5 × 2.4 × 4.5 cm involving the right dorsal nasal cavity, the right ethmoid, and sphenoid sinus as well as the nasopharynx. A strong enhancement was seen after intravenous contrast administration (*tumor).

**Figure 3:**
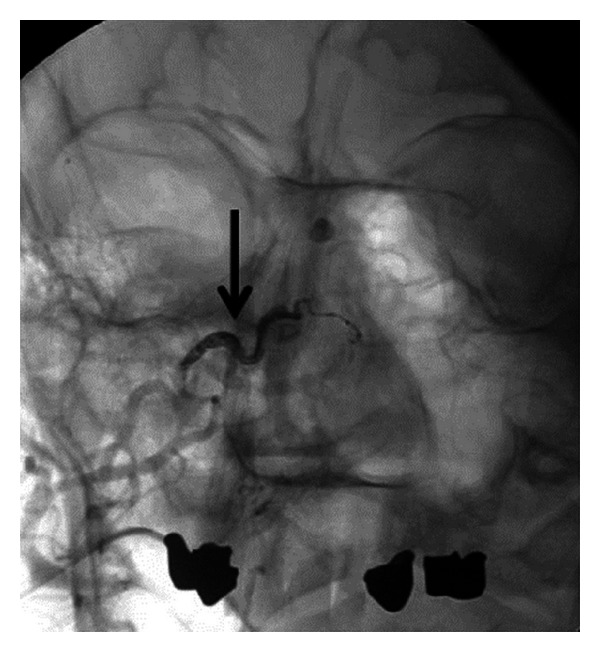
Sinonasal hemangiopericytoma after embolisation with Onyx. Right maxillary artery embolised with Onyx (→).

**Figure 4:**
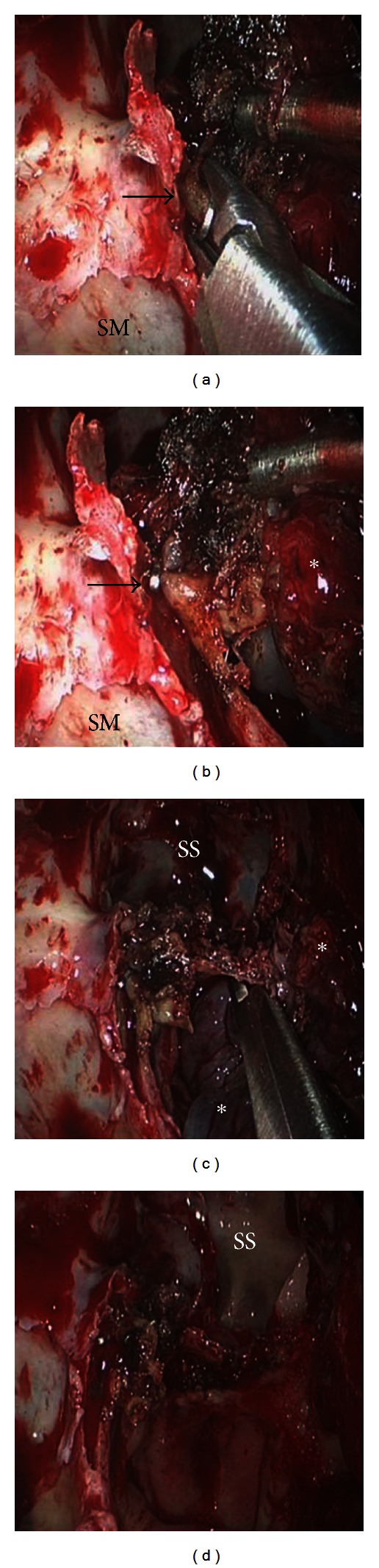
Endoscopically controlled resection. Operation started as a common functional endoscopic sinus surgery including clearing out the ethmoid cells and identifying the frontal sinus and the maxillary sinus. Upon resection of the dorsal part of the septum, a pedicled tumor insertion at the rhinobasis and the lateral nasal wall was found. The tumor's feeder vessels were exhibited. The vessels originating from the arteria sphenopalatine were specifically identified and clipped ((a), (b); →). The tumor was completely resected using four hands technique ((c), (d)). Maxillary sinus (SM), sphenoid sinus (SS), and tumor (*).

A 64-year-old Caucasian man in good general health presented to our institution complaining of nasal obstruction on the right side for 6 weeks. Further, the patient reported a singular episode of epistaxis. Other nasal symptoms such as recurrent epistaxis or sinusitis were absent. Head and neck examination revealed a polypoid mass obstructing the right nasal cavity subtotally (Figure 1(a)). The posterior rhinoscopia showed a partial obstruction of the nasopharynx as well. There was no cervical adenopathy or other disorder in the head and neck. A CT scan of the paranasal sinuses revealed a soft-tissue mass involving the right dorsal nasal cavity, the right ethmoid, and the sphenoid sinus as well the nasopharynx (Figure 2(a)). A strong enhancement was seen after intravenous contrast administration. Further, bone destruction of the right ethmoid structures was demonstrated. In the CT staging, neither local nor distant metastases were visible. The MRI confirmed a circumscribed, submucosal, heterogeneous mass measuring 5 × 2.4 × 4.5 cm, extending from the cribriform plate to the platinum durum, infiltrating the ethmoidal cells and the right maxillary and sphenoid sinus (Figure 2(b)). The cribriform plate also seemed to be infiltrated. The tumor was brightly vascularised with flow signal voids. Conventional digital angiography through the right external iliacal artery revealed a vascular network of the mass, mainly supplied by the right maxillary artery, as also seen in MRI angiography (Figure 2(c)). Additionally, the angiography showed a half-moon shaped blush, in particular of the dorsocranial portion of the tumor, supplied by ethmoidal branches of the right ophthalmic artery. A tumor resection by an endonasal approach was planned upon a biopsy revealing the suspicion of hemangiopericytoma. As the biopsy was followed by a prolonged bleeding and as the radiologic findings confirmed the high vascularisation of the mass, the right maxillary artery as the main vascular feeder was selectively embolised with an ethylene vinyl alcohol dissolved in the organic solvent dimethyl sulfoxide (Onyx) 24 hours before surgery (Figures 1(b) and 3). Following the preoperative embolisation, the endoscopic tumor resection was performed via endonasal approach with a navigation system. Upon resection of the dorsal part of the septum, a pedicled tumor insertion at the rhinobasis was exhibited in four hands technique and resected completely without any complication (Figure 4).

**Figure 5:**
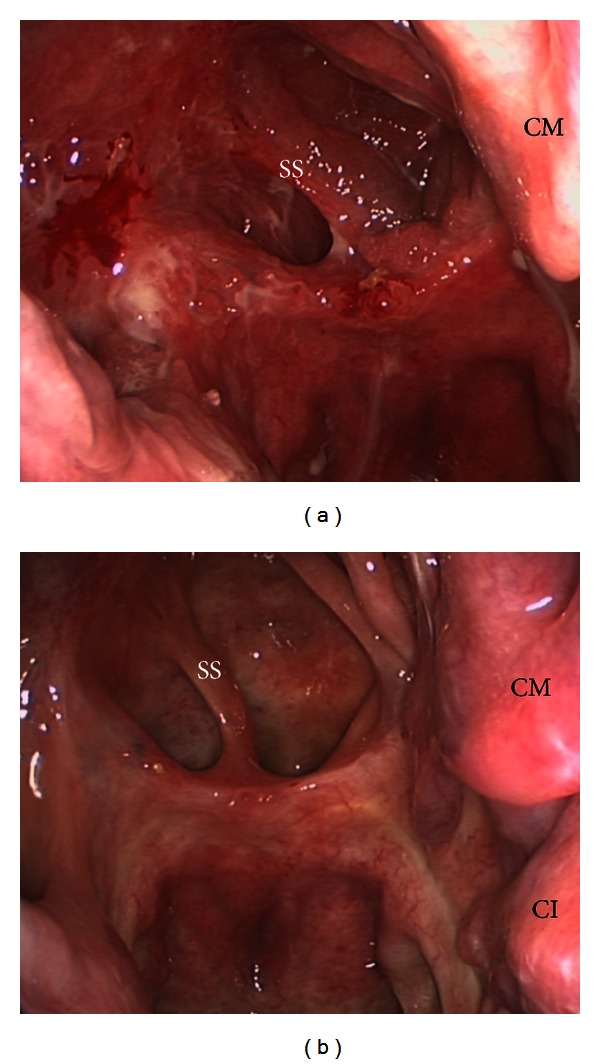
Endoscopic view of the operation field 3 months after surgery (a). No sign of recurrence. Twelve months after surgery (b), there is no sign of irritation. Concha inferior (CI), concha media (CM), and sphenoid sinus (SS).

**Figure 6:**
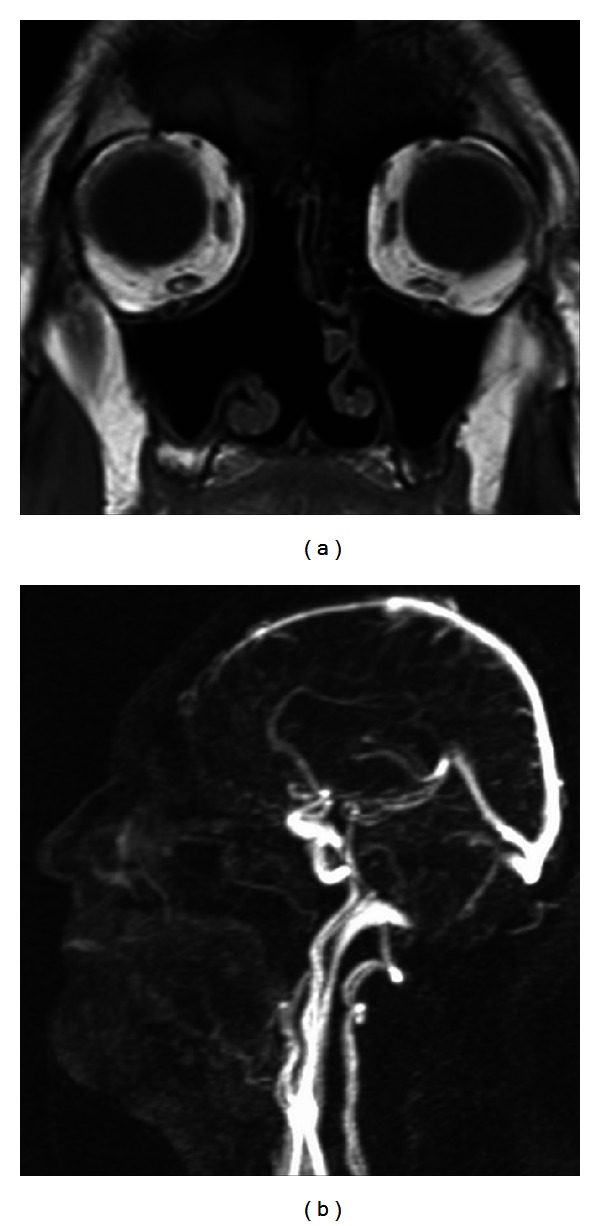
MRI (a) and MRI angiography (b) 12 months after surgery. The right nasal cavity appears to be free without any signs of tumor recurrence or residual.

Histologically, the tumor was submucosal, unencapsulated, and showed fascicular pattern. The tumor cells were uniform, spindle-shaped with oval nuclei, accompanied by an inflammatory cell infiltrate including eosinophils. “Stag horn” vessels, as typical for hemangiopericytoma, were focally identified as well as few nuclear atypia. Immunohistochemistry revealed a strong staining pattern to vimentin but no reaction with actin. The cells were immunoreactive for CD 34, slightly for Bcl2 and CD99 but negative for S-100 and epithelial membrane antigen (EMA). The Ki67-proliferation index was slightly increased at <2%. Due to a high recurrence rate of up to 25%, the patient has been followed up regularly by endoscopic surveillance and MRI controls. One year after surgery, there was no sign of locoregional disease recurrence, neither by endoscopy (Figures 5(a) and 5(b)) nor by MRI (Figures 6(a) and 6(b)).

## 3. Discussion and Literature Review

The term “sinonasal hemangiopericytoma,” formed 60 years ago, covers a wide range of neoplasia, all characterized by their histological appearance consisting of a uniform cell pattern of high cellularity and staghorn vessel formations. As a diversity of tumors meet this criterion, the term “sinonasal hemangiopericytoma” has become disputed. Some authors prefer the term “intranasal hemangiopericytoma-like tumors” [5]; other studies postulate a close relationship with soft tissue hemangiopericytoma justifying the term “sinonasal hemangiopericytoma” [1]. The WHO classification of head and neck tumors proposed in 2005 that sinonasal hemangiopericytoma should be named glomangiopericytoma due to their similarity with glomus tumors [6] especially as there are studies revealing a closer relationship of sinonasal hemangiopericytoma to glomus tumors than to hemangiopericytoma [7].

In the clinical-pathological routine, the practice has become accepted to use the term “hemangiopericytoma” for all tumors with hemangiopericytoma-like histology after exclusion of other tumor entities [8]—as done in this case report.

Sinonasal hemangiopericytoma mainly affects middle-aged patients [9]. Generally, the manifestation of a sinonasal hemangiopericytoma is possible at any age; our collected literature cases revealed the age of 18 years as the earliest age of onset [10]. Some studies report an equal-to-slight female predominance [11]; other authors deny any gender predilection [12]. In our literature review, including the case reports of 206 patients, the gender of patients was mentioned in 65 cases only. This group of 65 patients in total was composed of 32 male and 33 female patients—reflecting an equal gender distribution. 

The etiology is still unknown [13]. Former theories that trauma, long-term steroid use, arterial hypertension, and hormone imbalance might be predisposing risk factors for sinonasal hemangiopericytoma [14] are regarded as obsolete in the contemporary literature [15, 16].

In 1976, Compagno and Hyams provided the first detailed description and characterisation of the histological findings of sinonasal hemangiopericytoma [5]. Thus, sinonasal hemangiopericytoma presents hardly any nuclear polymorphisms with no hemorrhagia, no necrosis, nor any other characteristics found in malign hemangiopericytoma at somatic sites. Due to the typical histological characteristic of the uniform cell pattern of high cellularity with staghorn vessel formations, Compagno and Hyams formed the term “hemangiopericytoma-like” [3, 5]. However, the histological findings firstly described in 1976 by Compagno and Hyams do not allow to make an explicit histopathologic diagnosis.

Large vessels with staghorn configuration, which are also often cited as characteristic for sinonasal hemangiopericytoma, are neither specific nor entirely sensitive findings as they may be seen in many other soft-tissue spindle-cell neoplasia [17].

Since immunochemistry has found its entrance into pathology, several epitopes have been identified to help diagnosing sinonasal hemangiopericytoma. However, vimentin and CD34 are considered as the only antigens to be reliably detected in tumor cells of sinonasal hemangiopericytoma [12], whereas later studies state a lower CD34 expression of 80%–90% [8]. Other epitopes as actin, S-100 or factor XIIIa can be found in a few cases only [18]. Though, the histological detection of vimentin and CD34 does not allow any discrimination from other neoplasms of the entity of solitary fibrous tumors. According to recent studies, the vessels of the sinonasal hemangiopericytoma partially show a positive staining for D2-40 (the so-called podoplanin antibody). In case of positivity for D2-40, a clear discrimination between sinonasal hemangiopericytoma and other entities is possible, as podoplanin is not expressed by any solitary fibrous tumor other than hemangiopericytoma [8, 19].

The majority of sinonasal hemangiopericytoma is located in the nasal cavity itself and presents clinically with epistaxis and nasal obstruction [20, 21]. Pain occurs rather rarely and has to be regarded as sign of local infiltration [22]. Vision Impairment, headache, and local swelling are less frequent symptoms as well [23].

In the ENT examination, sinonasal hemangiopericytoma is frequently mistaken for inflammatory polpys [13]. Although only histology can find the final diagnosis [23, 24], biopsy is not recommended as it may lead to severe bleeding [20, 25]. Therefore, radiological examination by CT and/or MRI should preferentially be performed. A CT scan of the paranasal sinuses reveals a soft-tissue mass with strong enhancement after contrast administration and can clearly demonstrate bone destruction [26]. However, the CT scan does not allow a clear differentiation between tumor mass and inflammatory fluid in obstructed paranasal sinuses. Hence, it is highly recommended to perform an MRI scan as well. On T1-weighted MRI, sinonasal hemangiopericytoma appears as solid isointense masses with strong enhancement after intravenous contrast administration; on T2-weighted imaging, the tumor mass is isointense to low intense in contrast to inflammatory fluid reflecting high-intense signals [27]. The intravenous contrast administration during CT and MRI scan allows estimating the tumor vascularisation. The best visualisation of the vessel supply of the sinonasal hemangiopericytoma can be achieved by conventional digital angiography, which helps at the same time to plan a preoperative embolisation [23].

In order to estimate the risk of an aggressive clinical course, several criteria for malignance have been established. A large tumor size of >6.5 cm and the histological finding of necrosis, nuclear atypia, and a high number of mitosis are associated with a poorer prognosis [9]. However, the presence of one of these features does not predict an aggressive clinical course; it is rather the sum of several criteria for malignancy to be considered as prognostically unfavorable [1, 17]. Though, there are sinonasal hemangiopericytoma without any of the mentioned criteria which nevertheless develop malignancy. Hence, a certain potential of malignancy should always be taken into account [8].

In the meantime—concerning meningeal hemangiopericytoma—it has become apparent that metastases are the only proven sign of a poor outcome, whereas histological features fail to show any prognostic value [16, 28].

In contrast to hemangiopericytoma located at somatic sites, sinonasal hemangiopericytoma has a very low tendency for metastasis. The metastasis rate is approximately 5% [3]. However, lymphogenous and hematogenous metastases are described in the literature [14]. Regional lymph nodes as well as lungs, liver, and bone are the most common site of metastasis [11, 20].

Thus, the lethality of sinonasal hemangiopericytoma is less than 5%, in opposition to somatic hemangiopericytoma exhibiting a lethality of 25%–60% [3, 16].

In sinonasal hemangiopericytoma, the therapy of choice is indisputably the wide-field excision with negative margins [14, 29]. Only a few years ago, an external surgical approach, usually via lateral rhinotomy, was considered as the standard treatment regimen [22, 24] and was even regarded as obligatory when the tumor breached the cribriform plate [30] or spread beyond the sinonasal region [24]. An elective neck dissection is not indicated as lymphogenous metastases occur rarely [29].

Recently, the tumor resection via endonasal approach became more important in the treatment of sinonasal hemangiopericytoma. Out of 206 cases published in the last two decades and collected in this literature review, 57 patients received an endoscopic endonasal resection, of which 23 patients underwent a preoperative embolisation.

The endoscopic controlled endonasal approach offers many advantages compared to the external approach—particularly, a better overview for accurate assessment of the tumor insertion, the margins and the surrounding tissue. Obtaining a comparable view with an external approach would only be possible by a surgically extensive exposition, which appears less appropriate for a usually localized tumor [20]. Further, an external approach is associated with an increased amount of blood loss during the surgical exposure [31]. Other added benefits of an endonasal approach are the preservation of the natural physiology of the nose and the reduced risk of damaging the lacrimal structures. The avoidance of outer incision with consecutive scars is another advantage of the endoscopic controlled endonasal approach [20].

In very few cases only, an endoscopic controlled endonasal approach may be limited by certain factors, for example, a large tumor size with invasion in the fossa pterygopalatina, orbital involvement, or a highly vascularised tumor [20]. However, a high vascularisation of the tumor does not generally exclude an endoscopic resection provided that embolisation may be successfully performed before surgery. A preoperative embolisation helps to avoid intraoperative hemorrhagia and affects positively the tumor size and the area of resection [9].

Our personal experience let us to recommend an endoscopic controlled endonasal resection even in case of a large and highly vascularised tumor, preferably by using four hands technique in cooperation with a neurosurgeon. As condition precedent to an endonasal approach, we consider—apart from the surgeon's technical expertise—a successful embolisation before surgery. During endonasal endoscopic tumor resection, a proper hemostasis is crucial to avoid excessive bleeding necessitating a conversion to an external approach [24]. Twenty-three patients out of 206 collected literature cases of sinonasal hemangiopericytoma underwent an embolisation before surgery. However, no data are available concerning the embolisation with Onyx as successfully performed in the case hereby described.

Onyx is a liquid embolic agent, consisting of an ethylene vinyl alcohol dissolved in the organic solvent dimethyl sulfoxide (DMSO) with added tantalum powder for radiopacity. In the early 1990s, the use of a similar substance as an endovascular embolic agent for intracranial arteriovenous malformations was described by Taki et al. [32].

The polymerisation commences quickly upon the intra-arterial injection of Onyx. When placed in aqueous solutions or suspensions as blood, the DMSO diffuses out and causes the precipitation of the copolymer. Consequently, a spongy embolus which does not adhere at the delivery catheter is formed allowing a slow, controlled embolisation [33]. The depth of the distal vascular penetration by Onyx depends on the concentration of the copolymer providing a good handling of the embolic material [34].

The Onyx copolymer precipitates radially from the outer layer inwards over minutes to hours giving an excellent control throughout the embolisation with Onyx. Further, the Onyx embolus does not adhere to the delivery catheter. Therefore, the catheter does not have to be withdrawn as quickly as when using other liquid embolic agents, allowing a controlled and safe embolisation [35].

In the hereby described case of preoperative embolisation with Onyx, there were no adverse effects apart from a garlic similar smell during the first 36 hours after surgery, which is based on the expiration of the DMSO. Hence, Onyx is a safe and effective liquid embolic agent for the preoperative embolisation of sinonasal hemangiopericytoma.

To our knowledge, there are no prospective studies concerning the effectivity of other therapy modalities as radiotherapy or chemotherapy; all available data are based on retrospective studies with a rather small number of cases [20].

Chemotherapy shows a limited effectivity and is indicated as palliative treatment in the event of inoperable tumors or metastases [36]. Methotrexate, cyclophosphamide, vincristine and adriamycin may lead to a partial remission [37].

Radiotherapy as primary therapy of hemangiopericytoma of all sites has met with very limited success and is associated with a recurrence rate of 87.6% during the first five years [38] compared to a recurrence rate of 47% after tumor resection as primary therapy [15]. Hence, the radiotherapy is recommended for unresectable tumors and should be combined with chemotherapy [24].

In the event of recurrence of which the incidence is according to the literature from 9.5% to 50%, depending on the length of the followup, a reresection is considered as the treatment of first choice [39]. Although an adjuvant radiation may reduce the risk of recurrence, it is controversially discussed due to the good outcome after the reresection and the side effects of radiation of the head and neck [20].

The prognosis of hemangiopericytoma is not definitively predictable, neither by the clinical appearance nor by the histological findings. Recurrence may occur after a prolonged disease-free interval and has been even reported 26 years after tumor resection so that a regular, life-long surveillance of patients with hemangiopericytoma is mandatory [12, 15]. Further, recurrence frequently precedes the development of metastases after tumor resection [29].

## 4. Conclusion

In sinonasal hemangiopericytoma, an endoscopically controlled, endonasal tumor resection after preoperative embolisation proved to be an excellent treatment method. Together with the surgeon's technical expertise, the embolisation with Onyx allows a well-controlled endoscopically endonasal resection of even extended sinonasal hemangiopericytoma.
